# The complete chloroplast genome sequence of *Populus trinervis*, and a comparative analysis with other *Populus* species

**DOI:** 10.1080/23802359.2020.1775525

**Published:** 2020-06-16

**Authors:** Xia Liu, Chong Sun, Zheng Chen, Xia Gong

**Affiliations:** a College of Landscape Architecture and Life Science, Chongqing University of Arts and Sciences, Chongqing, China; b Sichuan Academy of Botanical Engineering, Sichuan, China

**Keywords:** *Populus trinervis*, chloroplast genome, phylogenetic analysis, genetic information

## Abstract

*Populus trinervis*, which is a unique plant for China. The complete chloroplast genome sequence of *Populus trinervis* was characterized from Illumina pair-end sequencing. The chloroplast genome of *P. trinervis* was 156,415 bp in length, containing a large single-copy region (LSC) of 84,805 bp, a small single-copy region (SSC) of 16,505 bp, and two inverted repeat (IR) regions of 27,554 bp. The overall GC content is 36.70%, while the corresponding values of the LSC, SSC, and IR regions are 34.5, 30.5, and 42.0%, respectively. The genome contains 131 complete genes, including 86 protein-coding genes (62 protein-coding gene species), 37 tRNA genes (29 tRNA species), and 8 rRNA genes (4 rRNA species). The Neighbour-joining phylogenetic analysis showed that *P. trinervis* and *P. hopeiensis* clustered together as sisters to other *Populus* species.

## Introduction

*Populus* is a genus of deciduous flowering plants, which was traditionally divided into six sections based on leaf and flower characters. *Populus trinervis* is grouped into *Populus* section *Tacamahaca*, and its leaves are 4–7 cm long, 2.5–5 cm wide. Being native to China, *P. trinervis* is found in Sichuan provinces. Given to both natural and artificial inter-specific hybrids, the classification of polar is very difficult. The genetic relationships and evolutionary history of poplar are still poorly investigated (Zheng et al. 2017; Hou et al. 2018). *Populus trinervis* plays an important ecological role in boreal and temperate forests, serving as wildlife habitats and watersheds; they can dominate riparian forests, but are ecologically adaptable. *Populus trinervis* has high intraspecific polymorphism, adaptability to different environments, combined with a relatively small genome size. Consequently, *P. trinervis* represents an excellent model for understanding how different evolutionary forces have sculpted the variation patterns in the genome during the process of population differentiation and ecological speciation (Neale and Antoine 2011). Moreover, we can develop conservation strategies easily when we understand the genetic information of *P. trinervis*. In the present research, we constructed the whole chloroplast genome of *P. trinervis* and understood many genome variation information about the species, which will provide beneficial help for population genetics studies of *P. trinervis*.

The fresh leaves of *P. trinervis* were collected from Sichuan (30°05′N, 102°54′E). Fresh leaves were silica-dried and taken to the laboratory until DNA extraction. The voucher specimen (SMQY001) was laid in the Herbarium of Chongqing University of Arts and Sciences and the extracted DNA was stored in the −80 °C refrigerator of the Key Laboratory of College of Landscape Architecture and Life Science. We extracted total genomic DNA from 25 mg silica-gel-dried leaf using a modified CTAB method (Doyle 1987). The whole-genome sequencing was then conducted by Biodata Biotechnologies Inc. (Hefei, China) with Illumina Hiseq platform. The Illumina HiSeq 2000 platform (Illumina, San Diego, CA, USA) was used to perform the genome sequence. We used the software MITObim 1.8 (Hahn et al. 2013) and metaSPAdes (Nurket al. 2017) to assemble chloroplast genomes. We used *P. tremula* (GenBank: NC_027425) as a reference genome. We annotated the chloroplast genome with the software DOGMA (Wyman et al. 2004), and then corrected the results using Geneious 8.0.2 (Campos et al. 2016) and Sequin 15.50 (http://www.ncbi.nlm.nih.gov/Sequin/).

The complete chloroplast genome of *P. trinervis* (GenBank accession number MT482538) was characterized from Illumina pair-end sequencing. T The complete chloroplast genome sequence of *P. trinervis* was characterized from Illumina pair-end sequencing. The chloroplast genome of *P. trinervis* was 156,415 bp in length, containing a large single-copy region (LSC) of 84,805 bp, a small single-copy region (SSC) of 16,505 bp, and two inverted repeat (IR) regions of 27,554 bp. The overall GC content is 36.70%, while the corresponding values of the LSC, SSC, and IR regions are 34.5, 30.5, and 42.0%, respectively. The genome contains 131 complete genes, including 86 protein-coding genes (62 protein-coding gene species), 37 tRNA genes (29 tRNA species), and 8 rRNA genes (4 rRNA species).

**Figure 1.**
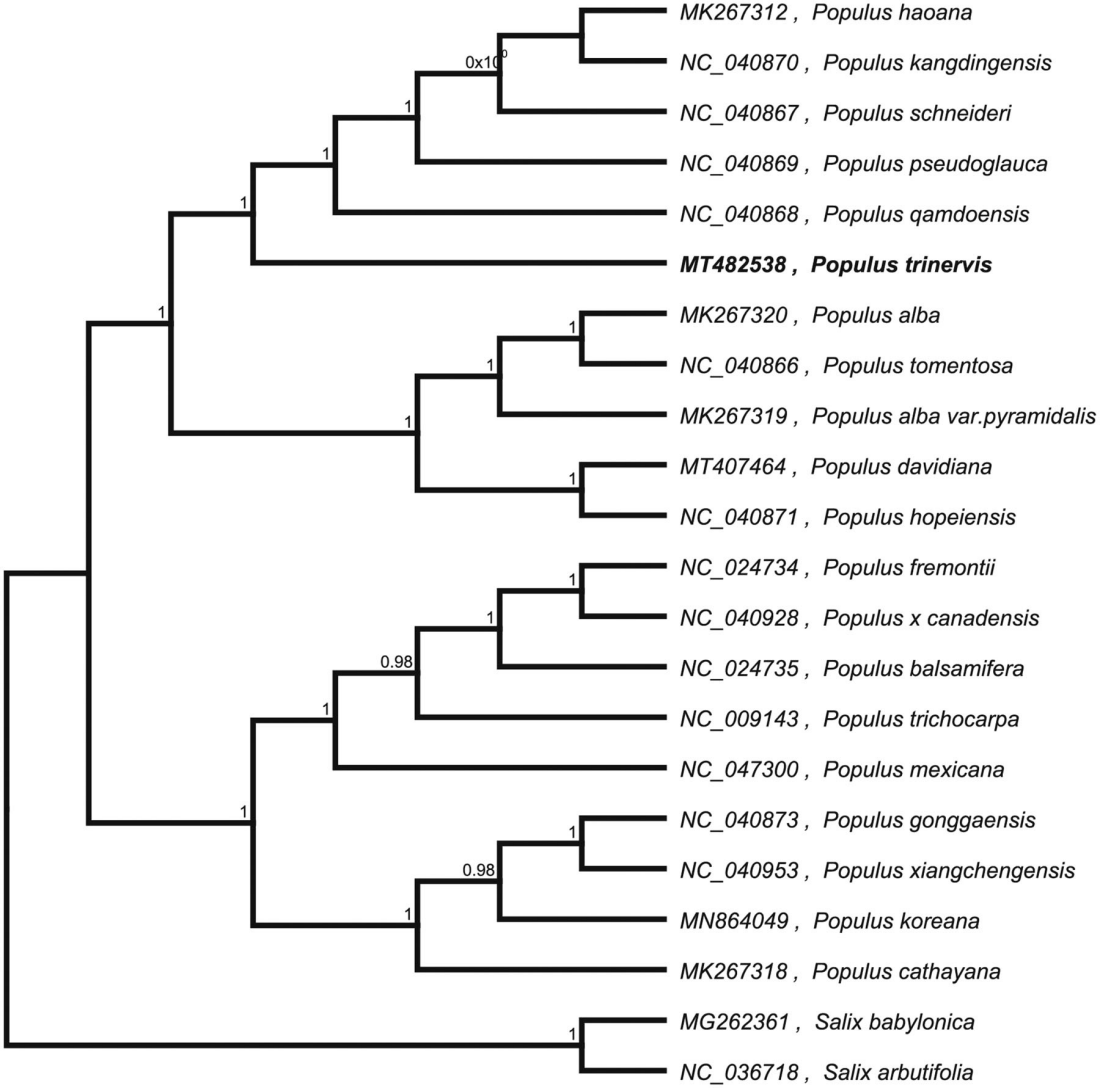
Neighbour-joining (NJ) analysis of *P. trinervis* and other related species based on the complete chloroplast genome sequence.

To confirm the phylogenetic location of *P. trinervis* within the family of *Populus*, we used the complete chloroplast genomes sequence of *P. trinervis* and 19 other related species of *Populus* and *S. babylonica* and *Salix arbutifolia* as outgroup to construct phylogenetic tree. The 14 chloroplast genome sequences were aligned with MAFFT (Katoh and Standley 2013), and then the Neighbour-joining tree was constructed by MEGA 7.0 (Kumar et al. 2016). The results confirmed that *P. trinervis* was clustered with *P. davidiana* (Figure 1).

## Data Availability

The data that support the findings of this study are openly available in National Center for Biotechnology Information (NCBI) at https://www.ncbi.nlm.nih.gov, accession number MT482538.
